# Willingness of female sex workers in Kampala, Uganda to participate in future HIV vaccine trials: a case control study

**DOI:** 10.1186/s12889-020-09932-7

**Published:** 2020-11-25

**Authors:** Yunia Mayanja, Andrew Abaasa, Gertrude Namale, Matt A. Price, Anatoli Kamali

**Affiliations:** 1 MRC/UVRI & LSHTM Uganda Research Unit, Plot 51-59 Nakiwogo Road, Entebbe, Uganda; 2 London School of Hygiene and Tropical Medicine, Keppel Street, London, WC1E 7HT UK; 3 IAVI, 125 Broad St, New York, NY 10004 USA; 4 Department of Epidemiology and Biostatistics, University of California San Francisco, 550 16th Street, San Francisco, CA 94143 USA

**Keywords:** Willingness to participate, HIV vaccine trials, Female sex workers, Sub Saharan Africa

## Abstract

**Background:**

We anticipate large efficacy trials of novel HIV vaccines that have shown acceptable safety profiles. We determined willingness to participate (WTP) in future HIV vaccine efficacy trials among HIV negative female sex workers (FSWs) in Kampala Uganda.

**Methods:**

We conducted a case control study in the Good Health for Women Project cohort. Cases received HIV prevention services and, enrolled in a 12-month simulated vaccine efficacy trial (SiVET) that used Hepatitis B vaccine; they underwent vaccine trial procedures as would be in an actual trial. Controls received similar health services but did not enroll in SiVET. We matched cases and controls (ratio 2:1) for age and duration in the cohort. We described a hypothetical HIV vaccine trial to cases (after 9 months in SiVET) and controls including trial attributes: randomization, delaying pregnancy, frequent blood draws (80-100mls) and study visits for 3 years. We compared WTP and willingness for vaccine trial attributes by case/control using chi-squared or Fisher’s exact tests and fitted conditional logistic regression models to determine independent predictors of WTP.

**Results:**

We analyzed data for 311 volunteers (219 cases, 92 controls); median age 27 years (IQR: 23–32), 39.9% had ≥secondary education, 57.9% had sex work as their main job and 81.9% used illicit drugs. Compared to controls, more cases had lived in the community for > 1 year, (85.4% vs 64.1%; *p* < 0.001) and fewer cases reported illicit drug use in the past 3 months, (79.0% vs 89.1%; *p* = 0.03). Overall, 278 (89.4%) volunteers expressed WTP in an HIV vaccine trial, the most common reason being hope of protection against HIV. More cases than controls (58.2% vs 44.7%) did not need to consult anyone before trial participation (*p* = 0.03); cases were more willing to delay pregnancy (99.0% vs 94.0%; *p* = 0.03). Combining vaccine trial attributes, 249 (89.6%) of the 278 accepted all attributes. After controlling for case/ control status women with secondary education or higher expressed less WTP (aOR 0.17; 95% CI 0.04–0.80).

**Conclusion:**

FSWs in Kampala demonstrated high WTP. Prior experience with trial requirements like contraception may improve their uptake during actual trials. Family involvement is important for those without prior trial experience.

## Background

Globally, the burden of new HIV infections in 2018 was 1.7 million a figure still far off the 2020 target of fewer than 500,000 new infections. Despite a global decline of 16% in the number of new HIV infections over the past decade, the number of people living with HIV, and HIV related deaths worldwide remain high [1]. In Sub-Saharan Africa (SSA), HIV prevention efforts need strengthening as the region still contributes the biggest proportion (64%) of the world’s new HIV infections, and 61% of HIV related deaths [1]. Combination prevention involving behavioural, biomedical and structural HIV prevention interventions is being scaled up [2]. It is evident that more biomedical HIV prevention technologies will be available in future as these are now at different stages in pre-clinical and clinical trials; some have already shown product efficacy and good retention in trials [3, 4].

However, novel biomedical HIV prevention technologies such as oral pre-exposure prophylaxis (PrEP), injectable PrEP and the anti-retroviral (ARV) vaginal ring require long-term adherence. Conversely, the HIV vaccine would not require long term adherence and is therefore more suitable for populations that have adherence challenges [5, 6]. Since the RV144 trial, HIV vaccines have been modified to give improved regimens that are safer with better-understood immunological responses and are now being tested for efficacy in the HVTN702 and HVTN705 trials [7, 8]. Results from these trials are eagerly awaited; they will provide data not only on vaccine efficacy but also optimal strategies for recruitment and retention in actual HIV vaccine trials.

Future HIV vaccine trials are likely to recruit huge sample sizes in order to demonstrate vaccine efficacy; ethics principles will require that volunteers receive proven interventions such as oral PrEP leading to an expected drop in HIV incidence [9]. The HIV vaccine being a more long term intervention is attractive to key populations in SSA including those that are highly mobile such as fisher folk and female sex workers (FSWs) [10, 11] who may not adhere well to short acting interventions that require frequent refills or clinic visits. These key populations tend to have a high HIV incidence and prevalence [1, 12–14] making them suitable potential volunteers in future vaccine efficacy trials, however their mobility may impact their adherence to more frequent trial procedures that require safety and other study outcome assessments. Scientists in the IAVI network in SSA have recently conducted Simulated Vaccine Efficacy Trials (SiVETs) to prepare suitable populations for future HIV vaccine efficacy trials. The SiVET studies, seen as important precursors to HIV vaccine trials mimic the rigors of HIV vaccine trials using licensed and available vaccines. In addition, they provide opportunities for study sites to develop and strengthen standard operating procedures and quality management systems that will be useful in the successful conduct of large efficacy trials. The success of future trials depends not only on site preparation and suitability of populations to show vaccine efficacy but also willingness to participate (WTP) in these trials. Given the limited data on WTP among FSWs, we determined WTP in future HIV vaccine trials among FSWs during the conduct of a SiVET that used hepatitis B vaccine (Engerix B) as a proxy for an HIV vaccine [15].

## Methods

### Study design

From July 2015 to April 2017, we conducted a case-control study among HIV negative women aged ≥18 years who were attending the Good Health for Women Project/ GHWP of MRC/UVRI and LSHTM Uganda Research Unit. It is located in a peri-urban community in southern Kampala and provides HIV prevention and treatment services to a cohort of FSWs. The choice of a case control study design was based on the need to investigate if willingness to participate in the future vaccine efficacy trials differed between FSWs that had participated in the simulation trial and those that had not. Cases comprised of volunteers who received clinic services and were enrolled from the GHWP cohort into a 12-month SiVET study designed to mimic the rigors of an HIV vaccine trial and administered a licensed recombinant Hepatitis B vaccine (ENGERIX-B™ GlaxoSmitheKline Biologicals Rixensart, Belgium). Controls comprised of volunteers who received the clinic services in the GHWP cohort but did not enroll in the SiVET. Cases and controls were matched for age and duration in the GHWP cohort in ratio 2:1. We selected controls from the GHWP database.

### Study population and sampling

We consecutively enrolled cases from the SiVET and their matched controls (non-SiVET volunteers-selected using simple random sampling if there were more than one matching control) attending the Good Health for Women Project (GHWP) clinic. The Good Health for Women Project (GHWP) clinic was established in a peri-urban community in southern Kampala in 2008. Women attending the clinic engage in sex with men for money, goods or favors and majority have attained primary level education. Vandepitte et al. have described recruitment of women from commercial hotspots [Vandepitte]. Women were enrolled into the GHWP clinic irrespective of HIV status, and invited for quarterly visits for HIV prevention, treatment and care services. At enrolment, they received HIV counselling and testing (HCT). HIV-positive participants received free HIV care including tuberculosis screening and treatment, prophylaxis for opportunistic infections and anti-retroviral therapy (ART). Repeat HCT was done at quarterly follow up visits only for women who previously tested HIV-negative. All women received free services including treatment for common illnesses, contraception, syndromic management of sexually transmitted infections (STIs), counselling for alcohol misuse, male and female condoms and treatment for their children below 5 years. They were encouraged to refer their male regular partners to the clinic for HIV prevention, care and treatment. Project field workers maintained contact details of enrolled participants. At the time of the SiVET study, the GHWP clinic had 2600 women actively attending the clinic quarterly for HIV prevention and treatment services of whom 290 were enrolled in the SiVET study.

### Sample size calculation

With 80% power and two-sided level of significance of 5% and a ratio of 2:1 (2-cases for each control, based on need to increase statistical power), we would detect 14% absolute difference in the proportion of participant willing to participate in the future vaccine efficacy trials between cases and controls. This difference was to be detected with precision of +/− 8%. Under these assumptions, a minimum of 280 cases and 140 controls were needed.

### Eligibility

We enrolled SiVET volunteers as cases if they attended the month 9 SiVET visit and were HIV negative. Volunteers who became HIV infected, pregnant or lost to follow up before month 9 were withdrawn from the SiVET. As they did not experience all trial attributes (e.g., completion of the vaccination schedule, delaying pregnancy) they therefore were not eligible to participate as cases in the WTP assessment. Controls were invited for the WTP assessment if they had an HIV negative test at the time of their GHWP clinic visit and were not participating in another study besides the GHWP cohort.

### Study procedures

We have previously described the SiVET study procedures among FSWs in Kampala [15], in which volunteers went through procedures as would be in an HIV vaccine trial.

#### WTP assessment for cases

At month 9 of follow up in the SiVET, trained study staff described a hypothetical HIV vaccine trial to volunteers who were eligible for the WTP assessment. They described vaccine trial attributes including: randomization (vaccine vs placebo), requirement to delay pregnancy during and 3 months after the vaccination phase, frequent study visits for 3 years, blood draws (80-100mls /8–10 tablespoons) and vaccine induced sero-positivity. The staff then used an interviewer-administered questionnaire to assess cases for WTP in such a trial if one ever took place. Those who missed the month 9 SiVET visit did the WTP assessment at month 12.

#### WTP assessment for controls

After the cases had been assessed for WTP, their matched controls who had been selected from the GHWP database were invited to receive the same hypothetical HIV vaccine trial information, information about vaccine trial attributes and complete the WTP assessment.

#### Vaccine trial attributes

Cases and controls who expressed WTP for future HIV vaccine trials after receiving hypothetical information were further assessed to determine willingness for the vaccine trial attributes.

### Study variables

The primary study outcome for this manuscript was willingness to participate in future HIV vaccine efficacy trials (WTP). WTP was documented it as a binary outcome *(Yes/No)*.

We selected independent variables based on literature review of studies that assessed WTP, and included socio-demographic variables as potential confounders.

The independent variables were education level, ethnicity, marital status, occupation; duration lived in the community and illicit drug use.

### Statistical methods

The study data was double entered in OpenClinica (version 3.1, USA) and analyzed in STATA 14 (StataCorp, College Station, TX, USA). We estimated the proportion of volunteers that expressed WTP stratified by case-control status as number that said they would accept participation divided by the total number of volunteers enrolled. We compared WTP among cases and controls using chi-squared tests or Fisher’s exact tests were applicable and fitted conditional logistic regression models to determine independent predictors of WTP. We further used counts and percentages to show the participants willingness to accept given vaccine attributes, compared cases controls using chi-squared or Fisher’s exact tests as necessary.

## Results

**Fig. 1:**
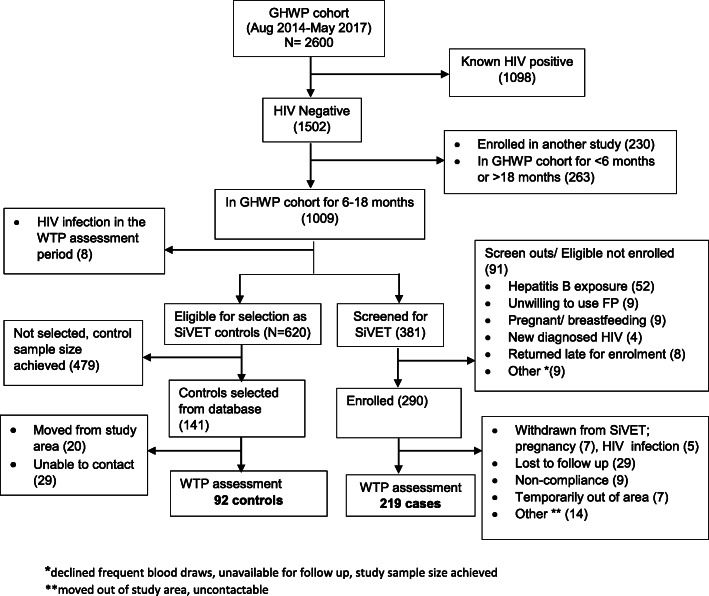
Screening Profile for volunteers assessed for willingness to participate in future HIV vaccine trials.*declined frequent blood draws, unavailable for follow up, study sample size achieved. **moved out of study area, uncontactable

Of 2600 women attending the GHWP cohort, we excluded 1098 (42%) known HIV positive, and HIV negative who were enrolled on another study (230, 9%) or had been in the cohort for < 6 months and > 18 months (263, 10%). Of the remaining 1009, 381 were screened for SiVET and 290 enrolled of whom 219 completed the WTP assessment while 92 of 141 selected controls completed the WTP assessment (Fig. 1).

### Baseline characteristics of study volunteers

We analyzed data for 311 volunteers (219 cases and 92 controls). Volunteer median age was 27 years (IQR: 23–32); cases 28 (24–34) and controls 25 (21–29), 39.9% had secondary education or higher, 57.9% had sex work as their main job and 81.9% used illicit drugs. The cases and controls did not differ by baseline socio-demographic characteristics except duration of time in the community (85.4% of cases compared to 64.1% of controls had lived in the community for > 1 year, *p* < 0.001) and use of illicit drugs in the past 3 months (79.0% of cases reported illicit drug use compared to 89.1% of controls, *p* = 0.03) (Table 1).

**Table 1:** Baseline Characteristics of FSWs assessed for WTP at the GHWP clinic in Kampala Uganda

Variable	Sub Category	Case (***n*** = 219)	Control (***n*** = 92)	***P***-value
Ethnicity	Baganda	113 (51.6)	45 (48.9)	0.256
	Banyankole	25 (11.4)	12 (13.0)	
	Banyarwanda	16 (7.3)	2 (2.2)	
	Other	65 (29.7)	33 (35.9)	
Education	Primary/None	126 (57.5)	61 (66.3)	0.149
	Secondary+	93 (42.5)	31 (33.7)	
Marital status	Single never married	55 (25.1)	25 (27.2)	0.704
	Married/ Single ever married	164 (74.9)	67 (72.8)	
Religion	Christian	164 (74.9)	72 (78.3)	0.525
	Muslim	55 (25.1)	20 (21.7)	
Duration (yrs) lived in community	0–1	32 (14.6)	33 (35.9)	**< 0.001**
	> 1	187 (85.4)	59 (64.1)	
Occupation	Other	95 (43.4)	36 (39.1)	0.489
	Sex work	124 (56.6)	56 (60.9)	
Illicit drug use in the past 3 months	No	46 (21.0)	10 (10.9)	**0.034**
	Yes	173 (79.0)	82 (89.1)	

### Willingness to participate in future HIV vaccine trials

Overall, 278 (89.4%) of women expressed WTP in an HIV vaccine trial after receiving hypothetical information and this did not vary by case or control status (89.0% vs 90.2%). The most common reason given for WTP was hope of protection against HIV (92.8% cases vs 92.8% controls). Both cases and controls did not consider volunteer reimbursements as a reason for WTP (0.6% cases vs 0% controls) and, 29.7% cases vs 55.4% controls expressed as reason for WTP to be among the first people for the vaccine to be tested on (*p* < 0.001). Of 33 volunteers (24 cases vs 9 controls) who did not express WTP, the main barriers were doubt about vaccine safety (20.8% cases vs 25.0% controls) and fear of vaccine-induced seropositivity (25.0% cases vs 12.5% controls).

When asked about the need to consult someone before trial participation, 58.2% cases vs 44.7% controls said they did not need to consult anyone (*p* = 0.03). The most common reason given for consultation was to seek knowledge about safety of the vaccine but this did not differ significantly between cases and controls (65.1% vs 55.6%). Other reasons given for consulting were to have support in case of side effects (25.3% cases vs 68.9% controls; *p* < 0.001) and to seek spousal consent (1.2% cases vs 13.3% controls; *p* = 0.008). Table 2 has details.

**Table 2:** Reasons for willingness and unwillingness to participate in future HIV vaccine trials

**Among those willing to participate in future HIV vaccine trials**	**Reasons**	**Case (*n*** **= 195)**	**Control (*n*** **= 83)**	***P*-value**
	Altruism-feels good to help	30 (15.4)	19 (22.9)	0.133
	To get the education about HIV	36 (18.5)	14 (16.9)	0.751
	To get the health care	78 (40.0)	38 (45.8)	0.371
	To get the regular HIV VCT	59 (30.3)	28 (33.7)	0.567
	Hope of being protected against getting HIV	181 (92.8)	77 (92.8)	0.988
	to be among the first people for the vaccine to be tested on	58 (29.7)	46 (55.4)	**< 0.001**
	to get some income	1 (0.6)	0 (0.0)	1.000
	Other	31 (17.2)	17 (21.0)	0.468
**Among those not willing to participate in future HIV vaccine trials**	**Reasons**	**Case (*n*** **= 24)**	**Control (*n*** **= 9)**	***P*-value**
	Anxiety/fear of catching HIV	4 (16.7)	0 (0.0)	0.191
	Concerns over blood draw	3 (12.5)	0 (0.0)	0.266
	Time commitment	3 (12.5)	1 (12.5)	0.913
	Fear of HIV positive result caused by the vaccine	6 (25.0)	1 (12.5)	0.385
	Feels has no risk of HIV, and therefore sees no need to be vaccinated	1 (4.2)	1 (12.5)	0.457
	Fears for the safety of the vaccine	5 (20.8)	2 (25.0)	0.931
	Other^a^	13 (54.2)	8 (88.9)	0.065

^a^Other (wants to conceive, transport challenges, compensation for time/travel not worth it, fear that participation, HIV status will be disclosed to others)

### Willingness for vaccine trial attributes

Among those who expressed WTP in future trials, willingness for different vaccine trial attributes was as follows: willingness for randomization as part of a clinical trial (99.6%), willingness to receive the candidate vaccine (99.2%), willingness to participate for 3 years was expressed (98.2%) and willingness to give large blood volumes of 80–100 mls (95.3%). Of 13 not willing to give large blood volumes, 12 volunteers expressed WTP for smaller blood volumes. We found a difference in the proportion of cases and controls willing to delay pregnancy (99.0% cases vs 94.0% controls; *p* = 0.03) (Table 3).

**Table 3:** Volunteer willingness for vaccine trial attributes stratified by case- control status

Variable	Category	Case (***n*** = 195)	Control (***n*** = 83)	***P***-value
Willing to participate for 3 years	Yes	192 (98.5)	81 (97.6)	0.637
	No	3 (1.5)	2 (2.4)	
Willing to provide 80–100 mls (8–10 tablespoons) of blood	Yes	184 (94.4)	81 (97.6)	0.357
	No	11 (5.6)	2 (2.4)	
Willing to be randomized	Yes	194 (99.5)	83 (100)	1.000
	No	1 (0.5)	0 (0.0)	
Willing to receive the candidate vaccine	Yes	193 (99.0)	83 (100)	1.000
	No	2 (1.0)	0 (0.0)	
Willing to use contraception during the vaccination phase and for 3 months after the last vaccination	Yes	193 (99.0)	78 (94.0)	**0.027**
	No	2 (1.0)	5 (6.0)	

Combining vaccine trial attributes, 249 (89.6%) of the 278 initially willing to participate found acceptable all the five vaccine trial attributes (randomization, trial participation for 3 years, blood draws of 80-100mls, requirement to delay pregnancy and receipt of the candidate vaccine). Twenty-six (9.4%) women, accepted four of the five vaccine trial attributes, two women accepted three attributes while one woman accepted only two. This did not vary by case control status, Fisher’s exact *p*-value 0.171.

After controlling for study arm (being a case or control) we only observed that women with secondary education or higher expressed less WTP (aOR 0.17; 95% CI 0.04–0.80).

## Discussion

Adult FSWs in Kampala demonstrated high WTP after receiving hypothetical HIV vaccine trial information. WTP was high irrespective of whether the women were SiVET volunteers (“cases” i.e. volunteers in a simulated trial designed to mimic the rigors of an HIV vaccine trial) or not (controls). Studies that have assessed WTP among other key populations in SSA and elsewhere have showed high WTP [16–19]. Reports however show that hypothetical willingness or motivation does not necessarily translate into participation in an actual HIV vaccine trial [20, 21]. Reported high WTP among volunteers who have not gone through or received education on vaccine trial procedures may therefore be due to responder bias. Cases had participated in the SiVET, a study done to mimic the rigors of an HIV vaccine trial, and at the time of the WTP assessment they had completed the vaccination phase and safety assessments of the SiVET study. The SiVET experience enabled volunteers to appreciate vaccine trial requirements and increased their confidence to make their own decisions to participate. Despite this, we found that their reported level of WTP did not vary significantly from that of the controls, who were followed in a cohort study with much less rigorous study procedures. This may suggest that participants of clinical research, at least in our case, are equivalently predisposed to participate in a hypothetical clinical trial, regardless of their current study commitment. The high proportion of WTP that we report could be explained by a high risk-perception for HIV infection among key populations and subsequent desire for protection against HIV.. Indeed, the most common reason women in our study gave for WTP was hope of protection against HIV as has been reported among FSWs in the US [18]. Findings from a SiVET study done among men who have sex with men (MSM) and FSWs in Kenya also indicate that higher perception of HIV risk is likely associated with WTP and other prevention behaviors like consistent condom use [19]. It is encouraging that key populations are willing to participate in future HIV vaccine trials, however it is also important for educations messages to emphasize that HIV vaccine candidates in future trials will be investigational products with unknown efficacy and not likely to offer total protection during the trial. HIV prevention interventions offered alongside HIV vaccine candidates would still have to be adhered to as detailed in trial procedures.

When we assessed vaccine trial attributes, WTP remained high for individual attributes but dropped slightly when we combined the five attributes. Cohort studies among Ugandan fisher folk that assessed similar vaccine trial attributes also, report lower proportions of WTP with combined vaccine trial attributes [16]. Although the proportion that expressed overall WTP did not differ between cases and controls, a higher proportion of cases than controls expressed willingness to use effective contraception in a future trial. Among fisher folk in Uganda women have reported high willingness to participate in future trials however, introduction of the vaccine trial requirement to delay pregnancy during and for a few months after the trial reduced WTP [16]. All women who attended the GHWP clinic received contraceptive services as part of health care [22] and they made a choice to use it. For the SIVET study however, willingness to use effective contraception was part of the study inclusion criteria [15] and, those not using contraception at SiVET study screening started on an effective method when enrolled. Cases were therefore more likely to use contraception compared to controls. In addition, women were using contraception for 9–12 months in SiVET before the WTP assessment; their experience of contraceptive use, related side effects and counselling could also have informed their higher willingness to delay pregnancy in future trials. Previous experience with some of the vaccine trial attributes such as use of contraception may increase their acceptability in future HIV vaccine trials.

Although both cases and controls expressed the need to consult (mainly spouse or parent) before making a decision to participate in a future vaccine trial, a significantly lower proportion of cases needed to consult someone. Our findings mirror the gender norms of society where a proportion of women still seek approval from family members before making decisions. It is therefore important to include significant family members such as spouses in the pre-trial community education meetings so that they appreciate trials, give their input into involvement of their family and community members and encourage those who express WTP. Furthermore, the lower proportion of cases that needed to consult could be because cases had lived longer in the community than controls and had a lower prevalence of illicit drug use. They were therefore more likely to be stable in the community and able to make their own decisions about future participation in trials. Follow up studies among fisher folk have shown higher retention rates among volunteers who live ≥5 years in a community compared to new entrants (< 1 year) [13].

Women with secondary education or higher expressed less WTP than those with lower education. A similar finding has been reported among different high risk groups in South India [23]. However Etchverry et al. report that higher education is associated with higher WTP among FSWs elsewhere [24]. It is expected that a higher education will enable better understanding of study education messages around HIV vaccine development and trials [25]. While Etchverry reports findings from a setting where FSWs are empowered, our study is done in a setting where sex work is still criminalized and FSWs discriminated [26–28]. Such structural barriers may negatively influence WTP in future HIV vaccine trials among FSWs with higher education levels who may also be more cognisant of the laws around sex work.

### Limitations and strengths

We determined WTP towards the end of the SiVET and therefore had cases who were willing to enroll and be followed up in clinical research. The controls were also attending the GHWP cohort for 3-monthly clinic visits for a duration similar to their matched cases. Both groups were thus highly motivated volunteers, and our estimates may not represent the WTP we might observe were we to do the same assessment in the cohort source population of FSWs in urban Uganda who have never participated in any clinical research. We recruited a smaller than expected sample size (88% of cases and 74% of controls) that could have reduced power to detect any differences between cases and controls. In addition, our results are based on hypothetical information; observed outcomes are prone to social desirability bias and may differ from what would be observed in an actual trial using a real HIV vaccine investigational product. One might hypothesize that those without the experience of a trial or simulated trial (i.e., the controls) might report a higher WTP, while the cases, with their greater experience, might report a lower, more “realistic” WTP. Not only did we not see a significant difference, but the reported WTP was very similar between groups (both around 90%). Since the SiVET mimicked the rigors of a vaccine efficacy trial, we believe that our results give more knowledge on WTP among FSWs and the significant differences between groups that participate in SiVETs compared to those who do not.

The WTP assessment looked at willingness to delay pregnancy and yet this was one of the inclusion criteria for the SiVET study. This led to inclusion in the SiVET of volunteers who were generally more willing to delay pregnancy in future trials and could have biased the assessment of this vaccine trial attribute between cases and controls.

## Conclusions

FSWs in Kampala are at substantial risk of HIV infection and are reportedly willing to participate in future HIV vaccine trials with similarly high willingness for different vaccine trial attributes. Involvement of family and/or community members is important and while they are needed for support with side effects, they will influence decisions of potential volunteers to participate in HIV vaccine trials. Previous experience with vaccine trial requirements such as contraceptive use is likely to improve uptake of the same during a real HIV vaccine efficacy trial.

## Data Availability

The datasets used and analyzed during the current study are available from the corresponding author on request.

## Abbreviations

ARV: Anti-retroviral
FSWs: Female sex workers
GHWP: Good Health for Women Project
HCT: HIV counselling and testing
HVTN: HIV Vaccine Trials Network
MRC/UVRI and LSHTM: Medical Research Council/ Uganda Virus Research Institute and London School of Hygiene and Tropical Medicine
PrEP: Pre-exposure prophylaxis
SiVET: Simulated vaccine efficacy trial
SSA: Sub-Saharan Africa
WTP: Willingness to participate
